# Truncated Total Least Squares Method with a Practical Truncation Parameter Choice Scheme for Bioluminescence Tomography Inverse Problem

**DOI:** 10.1155/2010/291874

**Published:** 2010-05-19

**Authors:** Xiaowei He, Jimin Liang, Xiaochao Qu, Heyu Huang, Yanbin Hou, Jie Tian

**Affiliations:** ^1^Life Sciences Research Center, School of Life Sciences and Technology, Xidian University, Xi'an 710071, China; ^2^School of Information Sciences and Technology, Northwest University, Xi'an, Shaanxi 710069, China; ^3^Institute of Automation, Chinese Academy of Sciences, Beijing 100190, China

## Abstract

In bioluminescence tomography (BLT), reconstruction of internal bioluminescent source distribution from the surface optical signals is an ill-posed inverse problem. In real BLT experiment, apart from the measurement noise, the system errors caused by geometry mismatch, numerical discretization, and optical modeling approximations are also inevitable, which may lead to large errors in the reconstruction results. Most regularization techniques such as Tikhonov method only consider measurement noise, whereas the influences of system errors have not been investigated. In this paper, the truncated total least squares method (TTLS) is introduced into BLT reconstruction, in which both system errors and measurement noise are taken into account. Based on the modified generalized cross validation (MGCV) criterion and residual error minimization, a practical parameter-choice scheme referred to as improved GCV (IGCV) is proposed for TTLS. Numerical simulations with different noise levels and physical experiments demonstrate the effectiveness and potential of TTLS combined with IGCV for solving the BLT inverse problem.

## 1. Introduction

In recent years, molecular imaging has emerged as a promising tool in basic, preclinical and clinical research for monitoring a variety of molecular and cellular processes in living organisms [1–4]. As one of molecular imaging modality, bioluminescence tomography (BLT) has attracted much attention due to its exquisite sensitivity and cost effectiveness.

The key problem of BLT is to reconstruct the bioluminescent source distribution inside a biological tissue from the optical signals detected on the body surface, which is a highly ill-posed inverse problem. By using numerical method such as finite element method (FEM), the inverse problem of BLT can be formulated into a nonsquare matrix equation, where the coefficient matrix is typically ill-conditioned [5]. Hence overcoming the ill-posedness and seeking a stable solution of the matrix equation are the major issues of BLT inverse problem. For this purpose, the inverse problem is often transformed to a least squares problem incorporated with the regularization technique. Tikhonov regularization is the most widely-used method in BLT reconstruction [6–8]. It is aiming to stabilize the inverse of an ill-conditioned operator and minimize the effects of the inevitable error by minimizing a trade-off between the loss function and the *l*_2_-norm [8]. However, previous studies based on Tikhonov regularization only consider noise in the measurement. In fact, some system errors also exist in the computed coefficient matrix of the system equation. These errors may take place in such aspects as FEM discretization, geometrical mismatch, optical parameters inaccuracy and model approximation, and so forth. System errors as well as measurement noise are inevitable in real BLT experiment, which may lead to large errors for the reconstruction results.

The total least squares (TLS) method is a generalization of the least squares approximation method when the data in both sides of the matrix equation are perturbed [9, 10]. Based on the TLS method, the truncated total least squares (TTLS) method is proposed for regularization of ill-conditioned linear systems [11]. It is inspired by truncated singular value decomposition (TSVD) which aims at limiting the contribution of noise by cutting off a certain number of terms in the singular value decomposition of coefficient matrix [12]. Truncation level plays the role of regularization parameter in truncation methods, which has great influence on the quality of the solution. As a result, determining an appropriate truncation level for TTLS is a critical step in the inverse procedure. Most existing parameter-choice schemes such as L-curve, discrepancy principle, generalized cross-validation (GCV), and zero crossing methods assume that the coefficient matrix is exactly known, that is, it is not contaminated by noises or errors [13–16]. In [17], a truncation level choice criterion named modified GCV (MGCV) is proposed for TTLS method; theoretical analysis and simulation tests show its potential for solving ill-posed linear system. However, it has been recognized that choice schemes of regularization parameter are mostly problem-dependent and practical parameter-choice scheme for BLT reconstruction deserves further study.

In this paper, the aim of our study is to extend the BLT reconstruction to the case including both the measurement noise and the system errors. For this purpose, TTLS method combined with a practical scheme termed as improved GCV (IGCV) is proposed to solve the BLT inverse problem. In the next section, our methodology of solving the inverse problem in BLT is described. In Section 3, we demonstrate the performance of the TTLS method combined with IGCV scheme in BLT reconstruction using numerical simulation and physical experiments in various source and noise level settings. Finally, we draw a conclusion and discuss the relevant issues.

## 2. Methodology

### 2.1. Diffusion Approximation and Boundary Condition

In general, light propagation in living subjects is mainly hindered by both tissue scattering and absorption [7, 8]. Considering that bioluminescent photons belong to the near-infrared region where scattering predominates over absorption [3], the propagation of photon can be well modeled by the following steady-state diffusion equation [18]:

(1) $$-\nabla \cdot \left(D\left(x\right)\nabla \Phi \left(x\right)\right)+\mu_{a}\left(x\right)\Phi \left(x\right)=S\left(x\right)\;\left(x\in \Omega \right),$$

where *Ω* ∈ *R* ^3^ is the bounded domain, Φ(**x**) represents the photon flux density, and *S*(**x**) denotes the energy density distribution of an internal bioluminescence source, *D*(**x**) = 1/(3(*μ*_*a*_(**x**) + *μ*_*s*_′  (**x**))) is the optical diffusion coefficient with *μ*_*a*_(**x**) being the optical absorption coefficient and *μ*_*s*_′(**x**) the reduced scattering coefficient, respectively.

Assuming that the BLT experiment is performed in a totally dark environment, the equation is subject to a Robin boundary condition [18]:

(2) $$\Phi \left(x\right)+2A\left(x;n,n^{\prime }\right)D\left(x\right)\left(v\left(x\right)\cdot \nabla \Phi \left(x\right)\right)=0\;\left(x\in \partial \Omega \right),$$

where ∂*Ω* denotes the boundary, *v*(**x**) represents the unit outer normal on ∂*Ω*, *A*(**x**; *n*, *n*′) ≈ (1 + *R*(**x**))/(1 − *R*(**x**)) and *R* ≈ −1.4399*n* ^−2^ + 0.7099*n* ^−1^ + 0.6681 + 0.0636*n*, *n *is the ratio of optical reflective index of the inner tissue to that outside the boundary, and *n*′ is close to 1.0 when the subject is in air [8]. In a bioluminescent imaging experiment, the measurable photon flux density on ∂*Ω* can be calculated by the following outgoing radiation [18]:

(3) $$Q\left(x\right)=-D\left(x\right)\left(v\left(x\right)\cdot \nabla \Phi \left(x\right)\right)=\frac{\Phi \left(x\right)}{2A\left(x;n,n^{\prime }\right)}\;\left(x\in \partial \Omega \right).$$

### 2.2. The Model of BLT Reconstruction

Based on (1), (2), and (3), the essence of the BLT reconstruction is to estimate the light source distribution inside the biological tissues from the measured flux on the surface, given the corresponding optical parameters of the tissues. In order to solve the BLT inverse problem, FEM was introduced to solve the diffusion equation in [8, 18–20] because of its capability to process volume with arbitrary geometries. After the discretization using FEM, the linear relation between the bioluminescence source intensity *S* and the photon flux density Φ can be expressed as the following matrix form:

(4) MΦ=FS,

where Φ and *S* are the collection of all the nodal values of the photon flux density and source density, *M* = *K* + *C* + *B*  is a positive-definite matrix, and *K*, *C,* and *B* are called the mass, stiff, and boundary matrix, respectively. The photon density Φ can be obtained from Φ = *M* ^−1^ *F* *S*. In fact, only partial photon on the boundary can be acquired in the BLT experiment, therefore, Φ can be partitioned into the measurable boundary data Φ^*m*^ and other immeasurable values Φ^*i*^, and thus the reconstruction of the bioluminescent source is to identify the unknown vector *S* from the photon flux density Φ^*m*^. According to the uniqueness theorem, the BLT solution is not unique in the general case [21]. Some prior information or constraints such as permissible area of source should be imposed on the unknown variables to obtain a meaningful reconstruction result. Considering the source permissible region, we can obtain the linear relation between the photon flux density Φ^*m*^ and the source energy density distribution *S* ^*p*^ in the light source permissible region, that is,

(5) $$AS^{p}=\Phi^{m},$$

where the coefficient matrix *A* is ill-conditioned and can cause severe numerical instabilities in the solution. Therefore, it cannot be directly solved using a simple least squares method.

### 2.3. Regularization

In order to obtain a stable solution, regularization methods are typically used for solving inverse problems [8, 22, 23]. The commonly used Tikhonov regularization method approximately solves (5) by converting it into the following minimization problem:

(6) $$\min\;{||AS^{p}-\Phi^{m}||}^{2}+\lambda {||S^{p}||}^{2},$$

where *λ* > 0 is a properly chosen regularization parameter. As a function of the regularization parameter, the solution of (5) is given by

(7) $$S^{p}={\left(A^{T}A+\lambda I\right)}^{-1}A^{T}\Phi^{m}.$$

However, the reconstructions with Tikhonov regularization method assume that the coefficient matrix *A* is exactly known and noises only exist in the measurement. The regularization solutions computed by (7) do not take system errors into account.

As mentioned in the introduction, TLS method is designed for the case that both sides of the matrix equation are subject to errors. BLT inverse problem can be stated with TLS formulation as follows:

(8) $$\underset{\tilde{A},{\tilde{\Phi }}^{m}}{\min\;}{\;||\left(A,\Phi^{m}\right)-\left(\tilde{A},{\tilde{\Phi }}^{m}\right)||}_{F}\;\;\text{subject}\;\text{to}\;\tilde{A}S^{p}={\tilde{\Phi }}^{m},$$

where ||·||_*F*_ denotes the Frobenius norm, $\tilde{A}$ and ${\tilde{\Phi }}^{m}$ are the error versions of *A* and Φ^*m*^, respectively, and (*A*, Φ^*m*^) is the augmented matrix that combines matrix *A* and vector Φ^*m*^ by using Φ^*m*^ as the last column of the new matrix. Based on the TLS method, TTLS method is proposed by Fierro in [11] for regularization of ill-conditioned linear systems. In TTLS, the redundant information in (*A*, Φ^*m*^), associated with the small singular values, is discarded and the original ill-conditioned problem is replaced with another appropriate and more well-conditioned problem. 

The TTLS algorithm used in this paper can be summarized as follows.

1. Compute the SVD of the augmented matrix (*A*, Φ^*m*^)
  (9) $$\left(A,\Phi^{m}\right)=\tilde{U}\tilde{\Sigma }{\tilde{V}}^{T}=\overset{n+1}{\underset{i=1}{\sum }}{\overset{̅}{u}}_{i}{\overset{̅}{\sigma }}_{i}{{\overset{̅}{v}}_{i}}^{T},$$
  where $\tilde{U}$ is *m*×(*n*+1), $\tilde{V}$ is (*n* + 1) × (*n* + 1), and ${\tilde{\text{U}}}^{T}\tilde{\text{U}}={\tilde{V}}^{T}\tilde{V}=I_{n+1},$ and $\tilde{\Sigma }$ is an (*n* + 1) × (*n* + 1) diagonal matrix with the singular values ${\overset{̅}{\sigma }}_{1}>{\overset{̅}{\sigma }}_{2}>\cdots >{\overset{̅}{\sigma }}_{n+1}$ on the diagonal.
2. Select a truncation parameter *k* ≤ min  (*n*, rank  (*A*, Φ^*m*^)).
3. Partition the matrix $\tilde{V}\in {\text{R}}^{\left(n+1)\times (n+1\right)}$ such that
  (10) $$\tilde{V}=\left(\begin{array}{cc} \begin{array}{c} {\tilde{V}}_{11}\\ {\tilde{V}}_{21} \end{array} & \begin{array}{c} {\tilde{V}}_{12}\\ {\tilde{V}}_{22} \end{array} \end{array}\right),\;{\tilde{V}}_{11}\in {\text{R}}^{n\times k},\;{\tilde{V}}_{22}\in {\text{R}}^{1\times (n+1-k)}.$$
4. Then the TTLS solution is given by
  (11) $$S_{\text{TTLS}}^{p}=-{\tilde{V}}_{12}{\tilde{V}}_{22}^{†}=-\frac{{\tilde{V}}_{12}{\tilde{V}}_{22}^{T}}{{||{\tilde{V}}_{22}||}_{2}^{2}}.$$

In fact, the aim of TTLS regularization is to appropriately identify an optimal truncation level, and then to construct a truncated solution that can capture the essential features of the unknown true solution, without explicit knowledge about the true solution and even without a priori knowledge about the magnitude of the noise in the data. For this purpose, truncation level *k* must be carefully determined.

### 2.4. Choice of the Truncation Parameter

MGCV criterion proposed by Sima in [17] makes use of the filter factor formulation of the TTLS solution proved in [11]:

(12) $$S_{\text{TTLS}}^{p}=\overset{n}{\underset{i=1}{\sum }}f_{i}\frac{{\overset{̅}{u}}_{i}^{T}\Phi^{m}}{{\overset{̅}{\sigma }}_{i}}{\overset{̅}{v}}_{i},$$

where the filter factor values

(13) $$f_{i}=\overset{k}{\underset{j=1}{\sum }}\frac{v_{n+1,j}^{2}}{{||V_{22}^{k}||}^{2}}\left(\frac{{\overset{̅}{\sigma }}_{i}^{2}}{{\overset{̅}{\sigma }}_{i}^{2}-\sigma_{j}^{2}}\right),\;i=1,\ldots ,n.$$

The property used for choosing the truncation parameter *k* is that when the parameter is greater than a certain crucial value, the TTLS solution is very sensitive to the noise or errors. Specifically, for small truncation level *k*, the filter factors with indices *i* = 1,…, *k* stay close to 1 and the filter factors with indices *i* = *k* + 1,…, *n* stay close to 0; when the truncation level gradually increases to a certain critical value, the filter factors with indices nearby *k* increase dramatically. It implies a way to identify the value of *k* where the filter factors change their steady behavior into erratic growth behavior. 

As for the regularization problem in BLT, the choice of regularization parameter with classical GCV is by means of minimizing the GCV function:

(14) $$G=\frac{{||AS_{\text{reg}}^{p}-\Phi^{m}||}^{2}}{{\left(\text{trace}\left(I-AA^{†}\right)\right)}^{2}},$$

where *A* ^†^ presents the pseudoinverse of *A*. With filter factors, the denominator can be computed by means of the following expression:

(15) $$\text{trace}\left(I-AA^{†}\right)=m-\left(n-p\right)-\overset{p}{\underset{i=1}{\sum }}f_{i},$$

where *p* is the rank of matrix ∑ with the singular values on the diagonal. We denote the sum of the filter factors of TTLS solution by enp_*k*_ as the effective number of parameters:

(16) $$\text{en}{\text{p}}_{k}=\overset{n}{\underset{i=1}{\sum }}f_{i}=\overset{n}{\underset{i=1}{\sum }}\;\overset{k}{\underset{j=1}{\sum }}\frac{v_{n+1,j}^{2}}{{||V_{22}^{k}||}^{2}}\left(\frac{{\overset{̅}{\sigma }}_{i}^{2}}{{\overset{̅}{\sigma }}_{i}^{2}-\sigma_{j}^{2}}\right).$$

According to the properties of filter factors mentioned above, for a *k* above a certain critical value, the filter factors for TTLS solutions with indices nearby *k* are larger than 1. A fact can be derived that enp_*k*_ is greater than *k* when *k* reaches this critical value, which is used to modify the above classic GCV function to suit the TTLS case. And then the MGCV criterion for TTLS is obtained

(17) $$G=\frac{{||AS_{\text{TTLS}}^{p}-\Phi^{m}||}^{2}}{{\left(m-\text{en}{\text{p}}_{k}\right)}^{2}}.$$

However, the regularization parameter directly identified by (17) may be not optimal for the specific BLT reconstruction problem. Inspired by L-curve method, we propose a hybrid scheme that combines MGCV with the minimization of the corresponding residual norm for regularization parameter choice. The IGCV scheme for TTLS is summarized in the following steps.

**Step 1**

Use the MGCV criterion to get an initial truncation parameter *k* and compute *k*_max_, where *k*_max_ ≤ *n* is the maximum *k* such that enp_1_ ≤ enp_2_ ≤ ⋯≤enp_*k*_max__ ≤ *m*; at the same time an array of GCV function values *G*(*i*) is obtained, *i* = [1, *k*_max_].

**Step 2**

For *i* : *k* ~ *k*_max_, find the local minimum points that satisfy the conditions: *G*(*i* − 1) > *G*(*i*) and *G*(*i* + 1) > *G*(*i*).

**Step 3**

For all the local minimum points, compute the residual error ||*A* *S*_TTLS,*i*_′ − Φ^*m*^||, where *S*_TTLS,*i*_′ is an approximation of the TTLS solution for a given truncation level *i*, that is, only the top 70% of the nodal values are kept for computation convenience, and then the final truncation parameter *k* = argmin ||*A* *S*_TTLS,*i*_′ − Φ^*m*^||.

Thus, a proper truncation parameter *k* for TTLS is sought according the above IGCV scheme.

## 3. Experiments and Results

The experiments implemented in this section are to test the performance of TTLS combined with IGCV for BLT inverse problem. To demonstrate the effectiveness of the proposed scheme, we compare the following reconstruction algorithms: Tikhonov method with classical GCV (Tik-GCV), TTLS method with MGCV (TTLS-M), and TTLS method with the proposed IGCV (TTLS-I). The parameter-choice scheme of Tikhonov method is different from that of TTLS method because MGCV and IGCV are specially designed for TTLS. A similar scheme, namely, classical GCV, is adopted in Tikhonov method for comparison convenience. The qualities of the reconstruction are assessed by the following quantitative indices: relative residual error (RRE), reconstructed location error, and reconstructed source power. Here, RRE is used to depict the extent of the solution fitting the measured data and is defined as ||*A* *S* ^*p*^ − Φ^*m*^||/||Φ^*m*^||. Absolute error (AE) of the reconstructed source location is used to describe the accuracy of the reconstruction, which is defined by$\sqrt{{\left(x_{i,r}-x_{i}\right)}^{2}+{\left(y_{i,r}-y_{i}\right)}^{2}+{\left(z_{i,r}-z_{i}\right)}^{2}}$ , where (*x*_*i*,*r*_, *y*_*i*,*r*_, *z*_*i*,*r*_) is the reconstructed center of each source and (*x*_*i*_, *y*_*i*_, *z*_*i*_) the actual center. Considering the ill-posedness of the BLT inverse problem, it is difficult to discriminate the influence of small source of high density and large one of low density [24]. So we prefer reconstructed source power compared with the actual value to source density for evaluating the quality of the reconstruction results. And the source power is estimated by computing the integral ∫*S*(**x**)*d* **x** of the source intensity over its support [25].

### 3.1. Numerical Simulation Verifications

**Figure 1:**
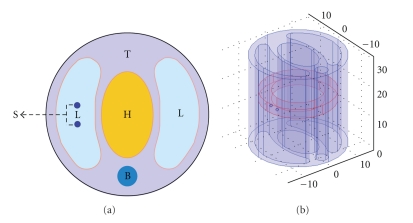
(a) A cross-section through two luminescent sources (S) in the left lung of a mouse phantom consisting of bone (B), heart (H), lungs (L), and tissue (T). (b) A 3D view of the permissible region.

In the numerical simulation, a 30 mm diameter and 30 mm high cylindrical mouse chest phantom is designed to evaluate the performance of the reconstruction method. The structure of the phantom is shown in Figure 1(a). The phantom is heterogeneous and the corresponding optical parameters are set as in Table 1 [25]. Two sphere sources of 0.5 mm diameter with 1 nW/mm^3^energy density are located in the left lung and the centers are *S*_1_ = (−9 mm, −1.5 mm, 15 mm) and *S*_2_ = (−9 mm, 1.5 mm, 15 mm), respectively. The power of each source is 0.5236 nW. In the following single source case, only the source centered at *S*_1_ is considered.

In order to reduce the ill-posedness of the inverse problem, a priori information of the source permissible region (PR) is incorporated to our method, which is shown in Figure 1(b) as PR = {(*x*, *y*, *z*) : 8 < (*x* ^2^ + *y* ^2^)^1/2^ < 12, 13.5 < *z* < 16.5} [25], where (*x*, *y*, *z*) is the coordinates of the corresponding FEM mesh vertices.

Generally speaking, simulated data used in reconstruction algorithms for inverse problems often come from the numerical solution of the forward problem. To avoid the typical issue of *inverse crime*, we use different FEM discretization for the forward process and reconstruction algorithms. Specifically, the forward model contains 11997 mesh vertices corresponding to 66334 tetrahedral elements, whereas the reconstruction model is consisting of 5277 vertices and 27465 tetrahedral elements. In addition, we employ Lagrange-Quadratic interpolation function in the forward process owing to the observation that high-order interpolation function can improve the numerical accuracy of the forward solution [26, 27].

To comprehensively simulate the noise and system errors involved in real BLT experiment, the photon flux density Φ^*m*^ is added with Gaussian white noise, and the coefficient matrix A is added with a system errors matrix. Due to the complexity of error sources, it is difficult to have an exact mathematic model to describe the system errors accurately. Hence we adopted the commonly used Gaussian white noise [28–30] and exponential noise to simulate the errors in matrix *A*, respectively.

**Figure 2:**
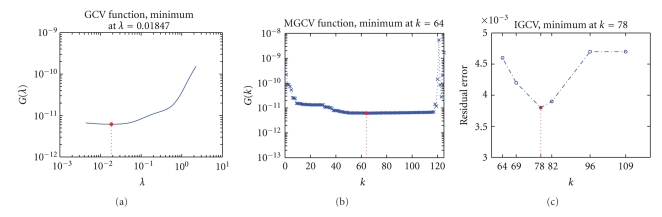
Regularization parameter determination in single-source case under measurement noise level of 10% and Gaussian system error level of 1%: (a) GCV function curve for Tikhonov, (b) MGCV function curve for TTLS, (c) illustration of the truncation parameter selection for TTLS with IGCV.

As discussed in Section 2, regularization parameter is the crucial factor that affects the quality of regularization solution to inverse problem. Figure 2 illustrates the determination of regularization parameters in single source case with measurement noise level of 10% and Gaussian system error level of 1%. Among them, Figure 2(c) shows the residual error values of all the local minimum points described in our improved scheme IGCV, which are used for the selection of an optimal truncation parameter *k* for TTLS. It should be noticed that the parameter* k* identified by MGCV is 64, whereas the optimal parameter *k* obtained by IGCV is 78. It is because ||*A* *S*_TTLS,78_′ − Φ^*m*^|| is 0.0038 and ||*A* *S*_TTLS,64_′ − Φ^*m*^|| is 0.0046, which indicate that 64 is not the optimal parameter value according to IGCV criterion. The determination of regularization parameter in double sources case is similar to that of single source case. For space limitation, we just provide the final regularization parameter obtained in various noise settings in Tables 2 and 3.

**Figure 3:**
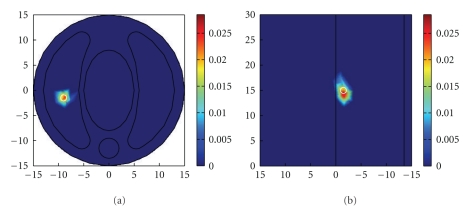
Reconstructed results under measurement noise level of 10% and Gaussian system error level of 1% with TTLS + IGCV: (a) *x*–*y* view of at *z* = 15 mm plane, (b) *y*–*z* view at *x* =−9 mm plane; the white circle indicates the real source.

In single source test, we found that all the methods under consideration can detect the source with the same center location *S*_1_^*R*^ = (−9.20 mm, −1.62 mm, 14.12 mm) in different noise levels, but the reconstructed source power varies with different reconstruction methods. Although the absolute error of the source location is 0.911 mm, the reconstructed source center is the nearest node to the original location in the aforementioned FEM discretization. Figure 3 only shows the reconstruction results by our proposed method with measurement noise level of 10% and Gaussian system error level of 1%. The detailed quantitative reconstruction results for the single-source model in various noise settings are listed in Table 2. The optimal results are listed in bold. Based on the simulation results in single-source case, it is clear that all the reconstruction methods can estimate the source location with no matter Gaussian or exponential noise in matrix *A*, but TTLS combined with IGCV performs best in all quantitative indices under different noise or error levels. 

**Figure 4:**
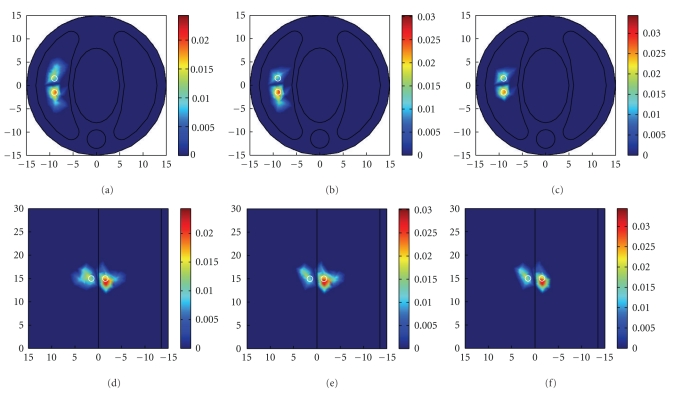
Reconstructed results in double source case under measurement noise level of 20% and system error level of 5%. (a), (b), and (c) separately show the *x*–*y* views at *z* = 15 mm plane of the results by Tikhonov + GCV, TTLS + MGCV, and TTLS + IGCV; (d), (e), and (f) are the corresponding *y*–*z* views at *x* = 9.5 mm plane of the reconstruction results, respectively; the white circle indicates the real source.

In the double sources case, both of the two sphere sources located in the left lung are tested. The final reconstruction results are listed in Table 3. Under all the noise conditions considered in this paper, the three methods can reconstruct the two sources at *S*_1_^*R*^ = (−9.20 mm, −1.62 mm, 14.12 mm) and *S*_2_^*R*^ = (−9.42 mm, 1.69 mm, 14.94 mm), which are 0.911 mm and 0.467 mm away from the actual ones, respectively. In fact, they are the nearest nodes to the original source locations under the FEM mesh used in our tests. However, with the increase of noise or error level, besides the optimal nodes *S*_1_^*R*^ and *S*_2_^*R*^, some artifacts appear in the reconstruction results, which are illustrated in Figure 4. Simulation results in double sources case further show that although there are differences between the results of different noise pattern in matrix *A*, similar conclusions can be obtained. As shown in Table 3, the reconstruction results of TTLS combined with MGCV are comparable to that of TTLS combined with IGCV when noise level is low; whereas with the increase of noise or error, TTLS combined with IGCV outperforms the other methods in all quantitative indices.

For BLT inverse problem, permission region is an effective way to regularize the solution by restricting the source distribution within a proper permissible region. In order to further test the proposed method, a ball shape permissible region of 10 mm in diameter is utilized, which is expressed as PR′ = {(*x*, *y*, *z*)∣((*x* + 7.5)^2^ + *y* ^2^ + (*z*−15)^2^)^1/2^ < 5, (*x*, *y*, *z*) ∈ Left lung}. The sources settings in this section are the same as the aforementioned double sources case. The source distribution in the ball permission region was reconstructed, and the results are summarized in Table 4. Considering that the different system error pattern has little effect on the reconstruction results in the foregoing simulations, we only add Gaussian noise to the system matrix *A* in this section. 

It is shown in Table 4 that TTLS combined with IGCV still performs best under all the noise levels in terms of RRE, reconstructed power and source location. Compared with the results in Table 3, the location accuracy for ball shape permission region PR′ is lower. For example, the largest deviation of the reconstructed position of *S*_1_ is up to 1.2 mm. It is clear that all reconstruction methods under consideration suffer from performance degradation with the relaxation of the permission region. However; the proposed method outperforms the other two methods and produces acceptable reconstruction results in our tests.

### 3.2. Physical Experiment Verifications

**Figure 5:**
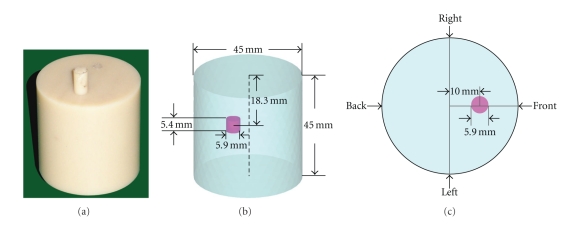
Physical phantom. (a) The homogeneous physical phantom; (b) The location of the single source in the phantom; (c) The cross-section of the phantom and the four directions of the CCD camera during data acquisition.

A physical experiment was carried out to further investigate the performance of the proposed method. A cylindrical phantom of 45 mm height and 22.5 mm radius was designed to evaluate different methods. The phantom shown in Figure 5(a) was made from nylon, and one small hole of 2.95 mm radius and 21 mm depth was drilled in the phantom to inject luminescent mixed solution used as the light source. In our physical experiment, the total volume of the mixed solution injected into the hole is 0.15 mL, thus a cylindrical source with a 2.95 mm radius and 5.4 mm height is centered at (9.88 mm, 1.5 mm, 26.7 mm), as shown in Figure 5(b). The optical parameters of the phantom were determined by a time-correlated single photon counting (TCSPC) system specifically constructed for the optical properties of the turbid medium [31]. The measured values of absorption and reduced scattering coefficients at the wavelength around 660 nm are 0.91 mm^−1^ and 0.0138 mm^−1^, respectively. 

**Figure 6:**
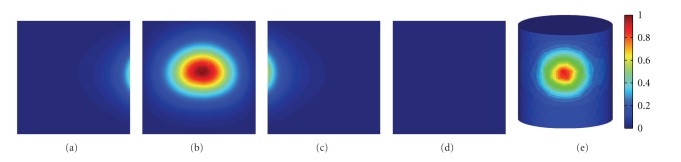
The normalized surface measurement of the homogeneous phantom. (a), (b), (c), and (d) are left view, front view, right view, and back view of the cylindrical phantom on the CCD camera, respectively; (e) is the flux density on the surface of the cylindrical phantom after mapping from the CCD camera.

A scientific cooled back-illuminated CCD camera (PIXIS 2048B) is used to collect the outgoing photons from the phantom surface. The photon flux density from different angles can be acquired by rotating the stage under the phantom, as illustrated in Figure 5(c). Figures 6(a)–6(d) exhibits the four views of the cylindrical phantom obtained by the CCD camera, respectively. Because the data captured by CCD camera is planar, mapping it onto 3D surface of the cylindrical phantom must be accomplished before reconstruction, which will also bring some inevitable errors to the measured data [32]. The mapping result was shown in Figure 6(e). 

**Figure 7:**
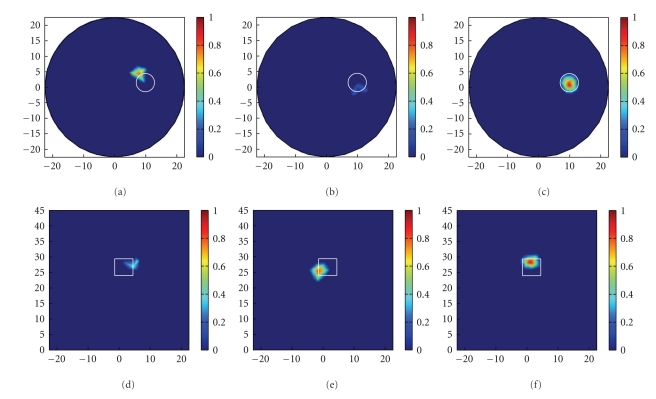
Reconstructed results in physical experiment: (a)–(c) are the *x*–*y* views at *z* = 28 mm plane of the reconstructed results using Tikhonov + GCV, TTLS + MGCV, and TTLS + IGCV method, respectively; (d)–(f) are the *y*–*z* views at *x* = 9.5 mm plane of the corresponding results, where the white contours indicates the real sources.

According to the photon flux density distribution on the phantom surface, the source permissible region is set as PR′′ = {(*x*, *y*, *z*) : 7 < (*x* ^2^+*y* ^2^)^1/2^ < 13, *x* > −3, 19.7 < *z* < 33.7}. In the reconstruction process, the phantom model consists of 2734 vertices corresponding to13551 tetrahedral elements. The schemes for the selection of regularization parameters are identical to those in numerical simulations. The final reconstruction results and the corresponding regularization parameter are listed in Table 5. The 3D views of the reconstructed results using different methods are presented in Figure 7, which verified the feasibility and effectiveness of the proposed method. As is evident from the images in Figure 7 and the data in Table 5, TTLS combined with IGCV successfully reconstructed the luminescent source with the minimum distance of 1.76 mm away from the actual source center.

## 4. Discussion and Conclusion

BLT reconstruction is a highly ill-posed inverse problem where small measurement noise and system errors in the input data can produce large changes in the results. In addition, bioluminescence signals are generally very weak, thus the noise or errors will significantly affect the reconstruction quality. Regularization technique has played an important role in solving BLT inverse problem. And most of the previous works assume that there is only measurement noise, which affects the right-hand side of the system equations. However, the computed coefficient matrix *A* in the model also has some errors, which may be caused by the calculation errors, the geometrical approximation, optical parameter inaccuracy, as well as the assumption of diffusion equation model itself. For example, the FEM discretization typically adds some errors to the matrix *A*. Hence, there is a need for seeking methods that can deal with the errors in both sides of the system equation. TTLS is a truncation regularization method that can take account of both system errors and measurement noise in the reconstruction process. This method depends on a parameter called truncation level; this single parameter has a significant influence on the regularization solutions. In this paper, IGCV, a practical scheme for determining the truncation parameter, is proposed to be combined with TTLS method for solving BLT inverse problem.

Simulations considering both system errors and measurement noise are conducted to investigate the performance of the proposed reconstruction method. Due to the lack of an accurate model to describe the system errors arising from multiple sources, commonly used Gaussian white noise and exponential noise are adopted to simulate the errors in matrix *A*, respectively. In addition, physical phantom experiments further test the proposed method.

Both the numerical simulations and physical experiments demonstrate the effectiveness of the proposed method. Tests with different noise levels show that TTLS with combined IGCV is able to produce much better reconstruction results than Tikhonov method, and TTLS combined with IGCV performs better than TTLS combined with MGCV, especially when both sides of the system equation are contaminated by measurement noise and system errors. Based on the experiments in this paper, we can draw a preliminary conclusion that TTLS combined with IGCV criterion is a potential reconstruction method for BLT inverse problem. Further investigation of the performance of the proposed method on animal experiments will be conducted in our future work.

## Figures and Tables

**Table 1:** Optical parameters of the heterogeneous phantom.

Material	*μ*_*a*_ (mm^−1^)	*μ*_*s*_′ (mm^−1^)
Tissue	0.007	1.031
Lung	0.023	2.000
Heart	0.011	1.096
Bone	0.001	0.060

**Table 2:** Quantitative results in single source case.

Sys. error	Meas. noise	Recon. method	Sys. error pattern	Regular. param.	RRE	Recons. power (nW)
Without errors	Without noises	Tik-GCV	N/A	0.00946	0.0263	0.4284
		TTLS-M	N/A	75	0.0243	0.4661
		TTLS-I	N/A	78	**0.0236**	**0.4797**
	10%	Tik-GCV	N/A	0.01437	0.0585	0.4096
		TTLS-M	N/A	69	0.0517	0.4535
		TTLS-I	N/A	78	**0.0419**	**0.5232**
	20%	Tik-GCV	N/A	0.02266	0.1083	0.3825
		TTLS-M	N/A	65	0.0809	0.4641
		TTLS-I	N/A	69	**0.0703**	**0.5214**

1%	10%	Tik-GCV	Gaus.	0.01847	0.0596	0.3502
			Exp.	0.02288	0.0705	0.3074
		TTLS-M	Gaus.	64	0.0590	0.3982
			Exp.	71	0.0562	0.3843
		TTLS-I	Gaus.	78	**0.0524**	**0.4389**
			Exp.	82	**0.0557**	**0.4113**
	20%	Tik-GCV	Gaus.	0.02844	0.1150	0.4295
			Exp.	0.02281	0.0897	0.3432
		TTLS-M	Gaus.	52	0.1142	0.4410
			Exp.	73	0.0599	0.4755
		TTLS-I	Gaus.	64	**0.0915**	**0.5427**
			Exp.	87	**0.0597**	**0.4829**

5%	10%	Tik-GCV	Gaus.	0.03771	0.0850	0.3069
			Exp.	0.05016	0.1023	0.2760
		TTLS-M	Gaus.	65	0.0823	0.3498
			Exp.	53	0.1018	0.2960
		TTLS-I	Gaus.	94	**0.0749**	**0.3553**
			Exp.	85	**0.0978**	**0.2995**
	20%	Tik-GCV	Gaus.	0.05414	0.1626	0.2722
			Exp.	0.06586	0.1871	0.2618
		TTLS-M	Gaus.	52	0.1536	0.3088
			Exp.	45	0.1758	0.2927
		TTLS-I	Gaus.	61	**0.1444**	**0.3359**
			Exp.	109	**0.1673**	**0.3044**

**Table 3:** Quantitative results in double source case.

Sys. error	Meas. noise	Recon. method	Sys. error pattern	Regular. param.	RRE	Recon. power (nW)	
						*S*_1_^*R*^	*S*_2_^*R*^
Without errors	Without noises	Tik-GCV	N/A	0.00858	0.0252	0.4671	0.2677
		TTLS-M	N/A	76	**0.0228**	**0.4824**	**0.2911**
		TTLS-I	N/A	76	**0.0228**	**0.4824**	**0.2911**
	10%	Tik-GCV	N/A	0.01533	0.0586	0.4334	0.2148
		TTLS-M	N/A	75	**0.0418**	**0.5047**	**0.3560**
		TTLS-I	N/A	75	**0.0418**	**0.5047**	**0.3560**
	20%	Tik-GCV	N/A	0.03001	0.1363	0.3633	0.1787
		TTLS-M	N/A	59	0.1074	0.4425	0.2069
		TTLS-I	N/A	71	**0.0739**	**0.5016**	**0.3004**

1%	10%	Tik-GCV	Gaus.	0.01711	0.0560	0.4164	0.2448
			Exp.	0.02148	0.0618	0.4174	0.1974
		TTLS-M	Gaus.	74	**0.0450**	**0.4581**	**0.3670**
			Exp.	71	**0.0534**	**0.5170**	**0.2440**
		TTLS-I	Gaus.	74	**0.0450**	**0.4581**	**0.3670**
			Exp.	71	**0.0534**	**0.5170**	**0.2440**
	20%	Tik-GCV	Gaus.	0.03112	0.1248	0.3506	0.1891
			Exp.	0.03034	0.1213	0.3688	0.1862
		TTLS-M	Gaus.	59	0.1023	0.4529	0.2042
			Exp.	50	0.1347	0.4085	0.2086
		TTLS-I	Gaus.	72	**0.0780**	**0.4768**	**0.2782**
			Exp.	58	**0.1159**	**0.4209**	**0.2253**

5%	10%	Tik-GCV	Gaus.	0.04179	0.0969	0.2613	0.2347
			Exp.	0.05154	0.1151	0.3528	0.2427
		TTLS-M	Gaus.	63	0.0890	0.3194	0.2647
			Exp.	50	0.1192	0.3495	0.2576
		TTLS-I	Gaus.	81	**0.0880**	**0.3399**	**0.2660**
			Exp.	65	**0.1141**	**0.3527**	**0.2906**
	20%	Tik-GCV	Gaus.	0.05077	0.1467	0.2859	0.1574
			Exp.	0.06730	0.1858	0.3122	0.2480
		TTLS-M	Gaus.	51	0.1376	0.3713	0.1574
			Exp.	48	0.1741	0.3557	0.3304
		TTLS-I	Gaus.	87	**0.1339**	**0.3692**	**0.2147**
			Exp.	54	**0.1698**	**0.3562**	**0.3306**

**Table 4:** Quantitative results for ball shape permission region  *P* *R*′ in double source case.

Sys. error	Meas. noise	Recon. method	Regular. param.	RRE	Recon. position (mm) and power (nW)			
					*S*_1_^*R*^		*S*_2_^*R*^	
1%	10%	Tik-GCV	0.02728	0.1720	(−8.80, −3.62,15.00)	0.2102	(−8.32,2.81,16.44)	0.2509
		TTLS-M	19	0.1535	(−8.80, −3.62,15.00)	0.2308	(−8.77,3.88,14.85)	0.2373
		TTLS-I	30	0.1523	(−8.80, −3.62,15.00)	**0.2978**	(−9.42,1.69,14.94)	**0.3062**
	20%	Tik-GCV	0.09397	0.4383	(−10.27, −2.24,16.42)	0.3633	(−10.97,2.08,16.69)	0.3501
		TTLS-M	9	0.4288	(−8.80, −3.62,15.00)	0.3782	(−8.77,3.88,14.85)	0.3651
		TTLS-I	28	0.4226	(−9.20, −1.62,14.12)	**0.5808**	(−8.32,2.81,16.44)	**0.4632**

5%	10%	Tik-GCV	0.09660	0.2350	(−10.27, −2.24,16.42)	0.3693	(−8.77,3.88,14.85)	0.3784
		TTLS-M	9	0.2350	(−8.80, −3.62,15.00)	0.3752	(−8.77,3.88,14.85)	0.3853
		TTLS-I	18	0.2171	(−8.80, −3.62,15.00)	**0.3899**	(−8.32,2.81,16.44)	**0.4234**
	20%	Tik-GCV	0.12804	0.4367	(−10.27, −2.24,16.42)	0.3549	(−8.77,3.88,14.85)	0.3578
		TTLS-M	15	0.3865	(−8.80, −3.62,15.00)	0.3900	(−8.77,3.88,14.85)	0.3653
		TTLS-I	19	0.3532	(−8.80, −3.62,15.00)	**0.4538**	(−8.77,3.88,14.85)	**0.3807**

**Table 5:** Reconstruction results in physical phantom experiment.

Recon. method	Regular. param.	RRE	Recon. source position (mm)	AE (mm)
Tik-GCV	0.00003	0.9513	(7.64,4.42,27.18)	3.71
TTLS-M	40	0.9015	(10.3, −1,19,25.50)	2.97
TTLS-I	45	**0.8318**	**(9.86,1.00,28.39)**	**1.76**

## References

[1] Willmann JK, van Bruggen N, Dinkelborg LM, Gambhir SS (2008). Molecular imaging in drug development. *Nature Reviews Drug Discovery*.

[2] Ntziachristos V, Ripoll J, Wang LV, Weissleder R (2005). Looking and listening to light: the evolution of whole-body photonic imaging. *Nature Biotechnology*.

[3] Tian J, Bai J, Yan X-P, Bao S, Li Y, Liang W, Yang X (2008). Multimodality molecular imaging: improving image quality. *IEEE Engineering in Medicine and Biology Magazine*.

[4] Contag CH, Bachmann MH (2002). Advances in in vivo bioluminescence imaging of gene expression. *Annual Review of Biomedical Engineering*.

[5] Schweiger M, Arridge SR, Hiraoka M, Delpy DT (1995). The finite element method for the propagation of light in scattering media: boundary and source conditions. *Medical Physics*.

[6] Tikhonov AN, Aresenin VY (1977). *Solutions of Ill-Posed Problems*.

[7] Wang G, Cong W, Durairaj K (2006). In vivo mouse studies with bioluminescence tomography. *Optics Express*.

[8] Lu Y, Zhang X, Douraghy A (2009). Source reconstruction for spectrally-resolved bioluminescence tomography with sparse A priori information. *Optics Express*.

[9] Huffel SV, Vandewalle J (1991). *The Total Least Squares Problem: Computational Aspects and Analysis*.

[10] Golub GH, Van Loan CF (1997). An analysis of the total least squares problem. *SIAM Journal on Numerical Analysis*.

[11] Fierro RD, Golub GH, Hansen PC, O’Leary DP (1997). Regularization by truncated total least squares. *SIAM Journal of Scientific Computing*.

[12] Hansen PC (1990). Truncated singular value decomposition solutions to discrete ill-posed problems with ill-determined numerical rank. *SIAM Journal on Scientific and Statistical Computing*.

[13] Hansen PC (1992). Analysis of discrete ill-posed problems by means of the L-curve. *SIAM Review*.

[14] Hansen PC, Kilmer ME, Kjeldsen RH (2006). Exploiting residual information in the parameter choice for discrete ill-posed problems. *BIT Numerical Mathematics*.

[15] Golub GH, Heath M, Wahba G (1979). Generalized cross-validation as a method for choosing a good ridge parameter. *Technometrics*.

[16] Moody JE, Moody (1992). The effective number of parameters: an analysis of generalization and regularization in nonlinear learning systems. *Advances in Neural Information Processing Systems*.

[17] Sima DM, Huffel SV (2007). Level choice in truncated total least squares. *Computational Statistics and Data Analysis*.

[18] Cong W, Wang G, Kumar D (2005). Practical reconstruction method for bioluminescence tomography. *Optics Express*.

[19] Song X, Wang D, Chen N, Bai J, Wang H (2007). Reconstruction for free-space fluorescence tomography using a novel hybrid adaptive finite element algorithm. *Optics Express*.

[20] Song X, Yi J, Bai J (2006). A parallel reconstruction scheme in fluorescence tomography basedon contrast of independent inversed absorption properties. *International Journal of Biomedical Imaging*.

[21] Wang G, Li Y, Jiang M (2004). Uniqueness theorems in bioluminescence tomography. *Medical Physics*.

[22] Cong AX, Wang G (2006). Multispectral bioluminescence tomography: methodology and simulation. *International Journal of Biomedical Imaging*.

[23] He X, Tian J, Wu Y, Hou Y, Ren N, Penga K Study of four regularization methods for the inverse problem in bioluminescence tomography.

[24] Dehghani H, Davis SC, Pogue BW (2008). Spectrally resolved bioluminescence tomography using the reciprocity approach. *Medical Physics*.

[25] Jiang M, Zhou T, Cheng J, Cong W, Wang G (2007). Image reconstruction for bioluminescence tomography from partial measurement. *Optics Express*.

[26] Hou Y, Tian J, Wu Y, Liang J (2009). A new numerical method for BLT forward problem based on high-order finite elements. *Communications in Numerical Methods in Engineering*.

[27] Han R, Liang J, Qu X (2009). A source reconstruction algorithm based on adaptive hp-FEM for bioluminescence tomography. *Optics Express*.

[28] Zhu W, Wang Y, Yao Y, Chang J, Graber HL, Harbour RL (1997). Iterative total least-squares image reconstruction algorithm for optical tomography by the conjugate gradient method. *Journal of the Optical Society of America A*.

[29] Zhu W, Wang Y, Zhang J (1998). Total least-squares reconstruction with wavelets for optical tomography. *Journal of the Optical Society of America A*.

[30] Shou G, Xia L, Jiang M, Wei Q, Liu F, Crozier S (2008). Truncated total least squares: a new regularization method for the solution of ECG inverse problems. *IEEE Transactions on Biomedical Engineering*.

[31] Qin D, Zhao H, Tanikawa Y, Gao F Experimental determination of optical properties in turbid medium by TCSPC technique.

[32] Ripoll J, Yessayan D, Zacharakis G, Ntziachristos V (2005). Experimental determination of photon propagation in highly absorbing and scattering media. *Journal of the Optical Society of America A*.

